# The Mediating Role of the Perceived COVID-19 Vaccine Benefits: Examining Israeli Parents’ Perceptions Regarding Their Adolescents’ Vaccination

**DOI:** 10.3390/vaccines10060917

**Published:** 2022-06-09

**Authors:** Shiran Bord, Carmit Satran, Ayelet Schor

**Affiliations:** 1 Health Systems Management Department, The Max Stern Yezreel Valley College, Yezreel Valley 1930600, Israel; schor.ayelet@gmail.com; 2 Nursing Department, The Max Stern Yezreel Valley College, Yezreel Valley 1930600, Israel; carmits@yvc.ac.il

**Keywords:** vaccine, hesitation, compliance, health belief model, adolescents, COVID-19

## Abstract

Israel was among the first countries to initiate adolescent COVID-19 vaccination. As adolescent vaccination requires parental consent, we evaluated the factors associated with parents’ willingness to vaccinate their adolescents and their point of view regarding adolescents’ involvement in this decision. An online survey was completed by 581 parents of adolescents aged 16–18. The main independent variables included trust in the healthcare system, components of the Health Belief Model (HBM) and adolescents’ involvement in the decision, as well as background data, including demographics. Analysis included a multiple logistic regression and mediation examination. Parents reported that 446 adolescents (76.8%) have been or will soon be vaccinated against COVID-19, 12.2% chose not to vaccinate their child and 11% have not yet decided. Vaccination was significantly associated with HBM components and with adolescents’ involvement in the decision. The perceived vaccination benefits acted as a mediator in the association between parents’ COVID-19 perceived threat and adolescent vaccination, as well as between parents’ trust in the healthcare system and adolescent vaccination. Addressing vaccination benefits and barriers is pivotal in the attempt to enhance adolescents’ vaccination adherence. Considering the importance of adolescents’ involvement in the decision, addressing them directly may also be beneficial in improving vaccination rates.

## 1. Introduction

On 20 December 2020, during the third lockdown in Israel, the country began vaccinating its population against the SARS-CoV-2 virus, thus, becoming one of the first countries to do so.

The updated data regarding worldwide vaccination rates indicate that adherence to COVID-19 vaccination varies among countries, and by March 2022, 64.4% of the world’s population had received at least one dose of a COVID-19 vaccine. In Europe, for example, vaccination rates range from 47.58% in Serbia to 92.6% in Portugal, while in the United States, vaccination rates have reached, approximately, 65.51%. In Middle-Eastern countries, adherence rates are among the lowest presented (Yemen 1.22%, Syria 7.22%, etc.), emphasizing the need to explore the political and social reasons for these low adherence rates [1]. In Israel, however, 6,128,853 people have been vaccinated twice (66.5% of the population), 4,476,373 three times (48.6% of the population) and 758,345 (8.2% of the population) have been vaccinated four times, as of the end of March 2022 [2].

The first to group get vaccinated in Israel were citizens over 60 years old. Gradually, additional age groups were added, until reaching the youngest adult group (20–40 years old). At the end of January 2021, Israel invited teenagers aged 16–18 to get vaccinated [2]. Adolescents’ vaccination was encouraged by the Israeli Ministry of Health (MOH), and like all ages, it was free of charge and performed in a widespread array of clinics and vaccination centers around the country.

While COVID-19 morbidity is less severe among children and adolescents [3,4], vaccinating them may assist in the attempt to reach a maximal rate of vaccination coverage and resume the pre-COVID routine. By the end of March 2022, 67.9% of Israeli adolescents in the relevant age group had been vaccinated [2]. Since children and adolescents are legally minors, their vaccination requires parental consent. Therefore, it is safe to assume that their vaccination rates depend on, among other things, parental attitudes and perceptions regarding the vaccine and its use. Vaccine hesitancy is a worldwide public health challenge. While vaccines are known to be a powerful tool in preventing illness and death caused by a vast range of pathogens [5], vaccine hesitancy is prevalent and vaccination adherence leaves a lot to be desired [6,7,8,9]. Insufficient vaccine adherence is routed in attitudes and perceptions, such as redundancy of the vaccine, poor threat perception regarding the relevant illness, low levels of trust in the healthcare system, etc. [10,11,12].

Parental hesitancy regarding children’s vaccination has been previously examined regarding various vaccines, such as Human Papillomavirus (HPV) and influenza. Barriers for HPV vaccination, for instance, found among hesitant parents included concerns regarding its novelty and long-term side effects [13]. The main concern expressed was related to the fact that it had not been sufficiently tested and, thus, there were not enough data regarding its safety and possible future side effects [14]. Further research aimed at identifying barriers to HPV vaccination found that a low perceived risk of HPV infection, concerns regarding the vaccine’s effect on sexual behavior, parental need for information, high perceived harm (from the vaccine), and low perceived vaccine effectiveness were among the leading factors presented [15]. In addition, health professionals’ recommendation was found to have a strong influence on parents’ decision to vaccinate their children in recommended childhood vaccinations [15,16,17,18]. As expected, adolescents living with HPV-vaccine-hesitant parents were less likely to receive the vaccine or complete the vaccine series [13].

Since COVID-19 vaccination is rather new, there is limited knowledge regarding the factors associated with responsiveness or hesitation to the vaccine among adolescents and children. A cross-sectional survey, which assessed parents’ willingness to vaccinate their children against the virus, found that 65% of parents intend to vaccinate their child when the vaccine becomes available [19]. An Israeli study conducted among Arab parents in Israel found lower rates of adherence, with only about 46% of the parents intending to vaccinate their children against COVID-19 [20]. Factors associated with parents’ willingness to vaccinate included the child’s age (adherence improved with age), a lack of chronic illnesses, and adhering to their routine vaccination schedule. The latter was also found to be a significant predictor in a survey aimed at assessing parents’ willingness to vaccinate their child against influenza [21]. A recent scoping review aimed at examining attitudes toward and influencing factors regarding COVID-19 vaccination of children and adolescents revealed further relevant aspects [22]. While it included a total of 34 studies, many of them examined vaccine responsiveness among children and adolescents without separating the different age groups and considering their characteristics [23,24,25]. According to this scoping review, children and adolescents’ COVID-19 vaccination acceptance rates vary from about 5% to 91% in the 34 studies reviewed [22]. The median acceptance rate was about 54%. The leading factors for vaccine acceptance were: worries about infection with COVID-19, mandatory vaccination policies, medical advice to get the vaccine, contribution to the control of COVID-19, beliefs regarding the effectiveness of the vaccine in protecting children, and beliefs regarding the vaccine’s safety and effectiveness. The perceived risk of their child getting sick with COVID-19 was a significant predictor of willingness to vaccinate in other studies as well [19]. The leading factors for hesitation were: medical reasons, the short-term protection of COVID-19 vaccines, the fact that children are at low risk for COVID-19 complications, lack of information and advice, worries about vaccine side effects, and worries about vaccine safety [20].

All these studies highlight the importance of the perceived disease threat, as well as the perceived benefits and barriers regarding vaccination. These factors are well described in the Health Belief Model (HBM) [26]. The model includes the individual’s perception of the disease threat, perception of the usefulness of performing the behavior (perceived benefits), and the perceived barriers for behaving in a certain way.

In addition to the factors described above, a Romanian study examining hesitancy regarding COVID-19 vaccination found social media to be a significant factor, affecting parental hesitancy [27].

Since the COVID-19 vaccine is novel and is based on new mRNA technology, not previously implemented to such an extent, trust also plays a pivotal role in the population’s acceptance of the vaccine. Previous studies have shown that increased trust in the healthcare system is associated with lower barriers and improved willingness to get vaccinated [28,29,30,31]. Moreover, distrust of governments and authorities predicts hesitancy and refusal to get vaccinated [32,33,34]. Numerous studies have demonstrated the importance of trust in the healthcare system and its association with compliance to both governmental recommendations and the COVID-19 vaccine among varied populations [31,35,36,37,38,39,40].

In addition to the parental role in affecting vaccination adherence, studies have found that adolescents’ attitudes and perceptions regarding vaccinations also play a significant role in the decision whether or not to be vaccinated [24,25]. An Italian study found that more than 40% of adolescents (aged 11–18) had very favorable attitudes towards the utility of vaccines in preventing disease. Moreover, almost half of the participants believed that adolescents should make vaccination decisions for themselves [14]. According to a recent Israeli study, 17.8% of adolescents said that getting the COVID-19 vaccine was their personal decision, and approximately 68% reported that it was a joint decision with their parents [41].

Considering the existing literature and as Israel was one of the first countries vaccinating adolescents, the country presents a unique opportunity to acquire knowledge regrading parents’ views and perceptions of COVID-19 vaccination, a novel vaccine in times of a world pandemic.

The main research question in the current study was: what are the factors associated with parents’ willingness to vaccinate their adolescents against COVID-19? This study aimed to address three main goals: (1) assessing the contribution of HBM’s components in parents’ willingness to vaccinate their adolescents against COVID-19; (2) examining the mediating role of the perceived COVID-19 vaccine benefits; and (3) investigating the adolescents’ role in the decision-making process (based on parents’ point of view). We hypothesized that: (1) HBM’s components will be associated with parents’ intention to vaccinate their adolescents; (2) the perceived COVID-19 vaccine benefits will play a significant role in parents’ willingness to vaccinate their adolescents; and (3) parents would consider their children’s perspective as relevant when contemplating vaccinating them against COVID-19.

## 2. Materials and Methods

The current study used an online cross-sectional research design, and was conducted from 3 February until 11 February 2021, a few days after the Israeli Ministry of Health invited adolescents aged 16–19 to get vaccinated against COVID-19. The survey was conducted by a large well-known online survey company (iPanel) with over 100,000 panel members. The sample aimed at representing the diversity of the Israeli Jewish population. To reduce possible selectivity bias, several reminders were sent to the prospective respondents. The study questionnaire was constructed based on a number of valid questionnaires in both Hebrew [37] and English [42,43]. The study questionnaire was translated into Hebrew and then back translated to English by an English editor. First, the co-authors examined face validity and intelligibility, then three experts examined the study questionnaire, providing expert validity. A pilot questionnaire was administered to ten parents, and as a result 2 items were better defined. Once improvements were made, the final questionnaire was formed.

### 2.1. Participants

The initial sample included 621 Jewish parents of adolescents aged 16–18. Nineteen parents reported that their adolescents were prohibited from vaccination due to medically related reasons, and 21 parents reported that their adolescents had been sick with COVID-19 and were, thus, ineligible for vaccination. These participants were excluded from the sample, which was, thus, composed of 581 parents, 414 mothers (71.3%) and 167 fathers (28.7%). The participants were 34–71 years old, with a mean age of 47.61 years (SD = 5.01). Most parents were married (*n* = 480, 82.6%) and had up to 13 children with a mean of 3.35 children (SD = 1.60). Most parents were Israeli born (*n* = 510, 87.8%), urban residents (*n* = 394, 68.0%), and secular (*n* = 399, 68.6%). Approximately half had an academic education (*n* = 305, 52.7%). Participants rated their income as above average (*n* = 251, 44.2%), average (*n* = 136, 23.9%), or below average (*n* = 181, 31.9%).

### 2.2. Measures

#### 2.2.1. Dependent Variable—Adolescents’ Vaccination against COVID-19

Parents’ willingness to vaccinate their adolescents against COVID-19 was measured by a dichotomous variable: has been or will soon be vaccinated against COVID-19 (1) vs. undecided or will not be vaccinated (0).

#### 2.2.2. Independent Variables

HBM Components—HBM subscales of perceived COVID-19 threat, barriers regarding vaccination, and benefits of being vaccinated were used [44,45]. Participants were asked to rate each item on a five-point Likert scale ranging from 1 (strongly disagree) to 5 (strongly agree).

1. Perceived COVID-19 threat

This was measured using three items regarding the perceived risk of COVID-19 to the adolescent and the perceived severity of COVID-19. Cronbach’s α was 0.70. Higher scores represented a higher perceived COVID-19 threat.

2. Perceived barriers regarding vaccination

Three items were used to measure barriers (including concern of vaccine safety and side effects). Cronbach’s α was 0.72. Higher total scores represented higher perceived barriers (e.g., “If my child is vaccinated against COVID-19, s/he might suffer from its complications”; “I am worried about the vaccination’s side effects”; and “I am apprehensive of the fact that the vaccination was developed in a hurry, with no information regarding its safety and quality control”).

3. Perceived vaccination benefits

These were measured using four items (Cronbach’s α 0.79). Higher total scores represented higher perceived benefits for vaccination (e.g., “If my child does not get vaccinated against COVID-19 this might block his/her access to many things, such as completing their high school diploma, driving lessons, etc.”; “Vaccinating my child against COVID-19 would allow him/her to resume his/her pre-COVID routine”).

#### 2.2.3. Trust in the Healthcare System

An eight-item instrument based on the Multidimensional Trust in Health-Care Systems Scale (MTHCSS) was used [42]. We used one item from the first subscale to measure trust in healthcare providers. In addition, we used all three items from the third and last subscale to measure trust in healthcare systems [37]. Participants were asked to rate each statement on a scale of 1 (strongly disagree) to 7 (strongly agree). Cronbach’s α was 0.84. Higher total scores represented greater trust in the healthcare system.

#### 2.2.4. Parental Compliance with the COVID-19 Guidelines (Parent Behavior)

Parental compliance was divided into two categories: (1) parents’ self-compliance with Ministry of Health’s (MOH) guidelines and (2) parents’ behavior relating to their adolescents’ compliance with MOH guidelines during the lockdown (e.g., “I have allowed my children to visit their friends at home”). The correlation between the two behavior variables was high (r = 0.50, *p* < 0.001); thus, they were averaged into one score and the higher the score, the more compliant the behavior.

#### 2.2.5. Parental Vaccination against COVID-19

This was measured using three categories: (1) yes, (2) plans to be vaccinated in the near future/was sick or is otherwise ineligible for the vaccination, and (3) does not intend to be vaccinated.

#### 2.2.6. Vaccination Routine among Children in the Family

Participants were asked to answer three questions regarding different vaccines their children had previously received:

1. Have your adolescents been vaccinated in the past with routine vaccines according to MOH guidelines? (1) Yes; (2) partly; (3) no. This variable was dichotomized into yes fully (1), vs. partly or no (0).
2. Do you vaccinate your children against seasonal influenza? (1—never, to 4—always). This variable wad dichotomized into: always or usually (1), vs. never or sporadically (0).
3. Have your children been vaccinated against HPV when administered in the 8th grade at school? (1) Yes; (0) no.

A total composite score was calculated, ranging 0–3, marking the adolescent’s vaccination status.

#### 2.2.7. Covariates

Parent—age, gender, country of birth, religiosity, marital status, number of children, place of residence, education, and income. A high correlation was found between level of education and income (r *=* 0.58, *p* < 0.001). Adolescents: age, gender.

In addition, participants were asked to respond with a yes/no answer to these questions:

“Have you been tested for COVID-19?”

“Have you been sick with COVID-19?”

“Has any family member been sick with COVID-19?”

Since most cases of parental sickness coincided with sickness of a family member, a total score marking parent/family sickness was developed (yes/no).

Finally, participants were asked to rate their assessment of their adolescent’s involvement in the decision to be vaccinated, using a ten-point Likert-type scale (“To what extent do you think the decision to get vaccinated depends on your son/daughter?”).

#### 2.2.8. Statistical Analysis

Analysis was conducted using IBM SPSS Statistics 27.0. Background characteristics were described using means and standard deviations, frequencies, and percentages. Internal consistencies were calculated with Cronbach α and variables were composed from item means. Simple logistic regression models were calculated to assess the extent to which each of the independent variables is associated with adolescents’ vaccination against COVID-19. Pearson correlations were calculated among the independent variables. A multiple logistic regression model was calculated to assess the extent to which the background variables, HBM components, and adolescent involvement were associated with adolescents’ vaccination against COVID-19. Mediation was examined with the Process Procedure Model 4 [46] for a binary outcome. Bootstrapping was used with 5000 samples and 95% confidence interval. The effect size f was used.

The reference population is Israeli adolescents, aged 16–18 years. Population size was 437,600 in 2020 [47]. According to Peduzzi et al. [48], the minimum sample size for a logistic regression in the current study was 474 participants.

## 3. Results

Most parents have been vaccinated against COVID-19 (*n* = 451, 77.6%) or reported that they intended to do so shortly (*n* = 28, 4.8%). Others had contracted the virus or were otherwise ineligible for vaccination (*n* = 40, 6.9%). Only about one-tenth reported that they did not intend to be vaccinated (*n* = 62, 10.7%). Most adolescents had received routine childhood vaccinations fully (*n* = 498, 86.2%) or partially (*n* = 58, 10.0%). Only a few had not (*n* = 22, 3.8%). Similarly, two-thirds of adolescents had been vaccinated against HPV (*n* = 383, 66.0%). However, numbers are different regarding influenza vaccinations: almost half of the adolescents seldom receive influenza vaccinations (*n* = 273, 47.1%) or receive them sporadically (*n* = 126, 21.7%), and a third receive them often (*n* = 101, 17.4%) or always (*n* = 80, 13.8%).

About three-quarters of the adolescents had been or will soon be vaccinated against COVID-19 (*n* = 446, 76.8%). Among these 446 adolescents, 76% (*n* = 339) had already been vaccinated and the rest (*n* = 107, 24%) will soon be vaccinated. Regarding the others, parents reported that they chose not to vaccinate their child (*n* = 71, 12.2%) or have not yet decided (*n* = 64, 11.0%). The likelihood of adolescent COVID-19 vaccination compliance was higher for older parents (OR = 1.05, *p =* 0.020, 95%CI = 1.01, 1.10, f *=* 0.013), for parents with fewer children (OR = 1.85, *p =* 0.009, 95%CI = 1.16, 2.94, f *=* 0.170), for parents with an academic education (OR = 1.48, *p =* 0.047, 95%CI = 1.01, 2.19, f *=* 0.108), for parents with an above-average income (OR = 1.64, *p =* 0.016, 95%CI = 1.10, 2.45, f *=* 0.136), and for adolescents who had received all their childhood and seasonal influenza vaccinations (OR = 2.29, *p* < 0.001, 95%CI = 1.81, 2.91, f *=* 0.228). The likelihood of adolescent vaccination was not associated with the responding parent’s gender (*p =* 0.068), with him/her being secular (*p =* 0.063), with the parent or a family member contracting the COVID-19 virus (*p =* 0.556), the adolescent’s gender (*p =* 0.179), or his/her age (*p =* 0.702). The likelihood of adolescent vaccination against COVID-19 was significantly associated with all HBM components, so that they were higher with a higher perceived threat and perceived benefits, and with lower barriers (threat: OR = 2.37, *p* < 0.001, 95%CI = 1.91, 2.95, f *=* 0.238; benefits: OR = 9.26, *p* < 0.001, 95%CI = 6.22, 13.79, f *=* 0.613; barriers: OR = 2.86, *p* < 0.001, 95%CI = 2.22, 3.57, f *=* 0.290). Moreover, they were higher for parents with a higher level of trust in the healthcare system (OR = 1.96, *p* < 0.001, 95%CI = 1.65, 2.33, f *=* 0.185), for parents with a more compliant behavior with the Israeli MOH behavior guidelines during the pandemic (OR = 1.35, *p =* 0.012, 95%CI = 1.07, 1.70, f *=* 0.083), and for greater reported adolescent involvement in the decision to be vaccinated (OR = 1.08, *p =* 0.009, 95%CI = 1.02, 1.15, f *=* 0.021).

Table 1 presents the distribution of the study’s independent variables and their intercorrelations. Parent’s age, number of children, and level of education were used as demographic control variables, due to their significant association with adolescent vaccination (income was not used due to its high correlation with level of education and as it included missing data).

As shown in Table 1, receiving childhood vaccinations was associated with a higher trust in the healthcare system, as well as with a higher perceived threat of the virus, higher benefits of being vaccinated, and lower barriers for vaccination. A higher trust in the healthcare system was associated with more compliant parent behavior with MOH guidelines, as well as with higher perceived threat, higher perceived benefits, and lower barriers for vaccination. Furthermore, compliant parent behavior was associated with the higher perceived threat of COVID-19 and higher perceived benefits of being vaccinated. Finally, higher perceived benefits of being vaccinated were associated with higher perceived threat and lower barriers.

Multiple logistic hierarchical regression was calculated to assess the extent to which the study variables are associated with the likelihood of adolescent vaccination (Table 2). Independent variables included background variables as well as contracting the virus in the family, variables representing conformity of perception and behavior, HBM components, and the adolescent’s involvement in the decision-making process.

The logistic regression model was found to be significant, explaining approximately 58% of the variance. The likelihood of adolescent vaccination was lower for parents with more children in the family (OR = 0.41, 95%CI = 0.19, 0.89, f *=* 0.246) and higher perceived barriers (OR = 0.49, 95%CI = 0.35, 0.70, f *=* 0.197). It was higher with higher compliance with childhood vaccinations (OR = 1.41, 95%CI = 1.01, 1.95, f *=* 0.095), perception of higher benefits of being vaccinated (OR = 6.42, 95%CI = 3.85, 10.69, f *=* 0.513), and greater adolescent involvement in the decision (OR = 1.09, 95%CI = 1.01, 1.18, f *=* 0.024). Clearly, the contribution of the HBM components is the highest, with that of vaccination benefits being higher than all.

**Figure 1:**
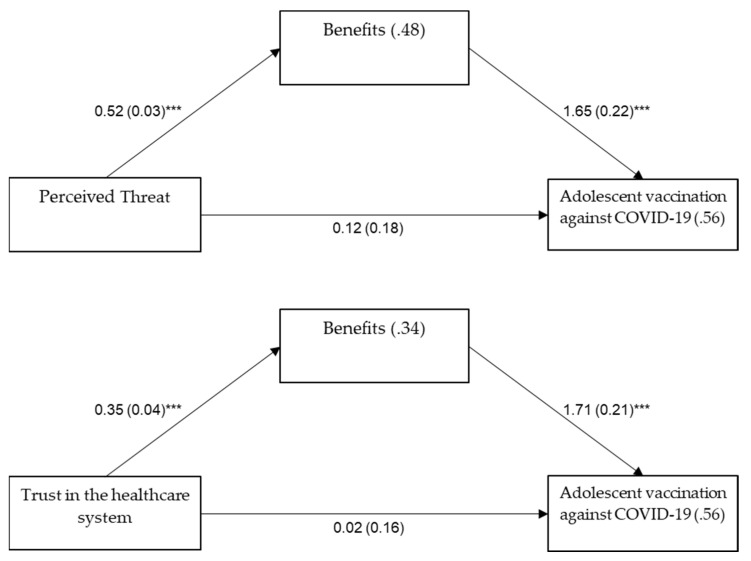
The mediating role of perceived benefits of vaccination in the association between perceived threat and trust in the healthcare system and adolescent vaccination for COVID-19. Note: values on arrows: B(SE), values within rectangles: R^2^ or Nagelkerke’s R^2^. *** *p* < 0.001.

Finally, the perceived benefits of vaccination were examined as mediating the relationship between the extent of threat that the virus poses, as well as trust in the healthcare system, and adolescent vaccination against COVID-19. The examination was calculated with the Process Procedure Model 4 for a binary outcome [46], controlling for parent age, number of children in the family, parent education level, COVID-19 sickness in the family, childhood vaccinations, and the barriers to vaccination. Mediation was found significant for both perceived threat (coefficient = 0.86, SE = 0.14, 95%CI = 0.65, 1.19) and trust (coefficient = 0.61, SE = 0.13, 95%CI = 0.42, 0.91) (Figure 1).

Perception of a higher threat, as well as higher trust in the healthcare system, were associated with perception of higher benefits of being vaccinated, which was then associated with higher odds for adolescent vaccination.

## 4. Discussion

Israel was one of the first countries vaccinating adolescents, thus, presenting a unique opportunity to acquire knowledge regrading parents’ views and perceptions of COVID-19 vaccination, a novel vaccine in times of a world pandemic.

Overall, Israeli parents’ willingness to vaccinate their adolescents against COVID-19 was very high. This may be due to an overall high level of knowledge regarding COVID-19 among the general adult population [49]. The likelihood of adolescents’ vaccination was higher with higher compliance with childhood vaccination, higher perception of vaccination benefits, lower perception of barriers regarding vaccination, and greater adolescent involvement in the decision-making process.

Previous studies have shown the association between routine childhood vaccination compliance and compliance with a new vaccine uptake. For example, adolescent girls that did not complete the recommended childhood vaccination plan on schedule were at higher risk of HPV vaccination non-compliance [50]. This may point to populations at risk of new vaccination non-compliance.

In concurrence with the HBM [26], our findings highlight the contribution of the HBM components, with that of the perceived benefits of vaccination being the highest. In the current study, the main perceived benefit of vaccination was the adolescents’ opportunity to resume their pre-COVID-19 routine, while the main barriers related to the vaccine’s novelty and its long-term implications. While these findings are consistent with those of previous studies, emphasizing the relevance of the HBM variables for vaccination adherence in general [18,44,50], and of the perceived barriers for vaccination specifically [15,17,21,51,52,53], the current study is novel in emphasizing the pivotal role of the perceived vaccination benefits. In addition, parents with higher levels of trust in the healthcare system were more inclined to perceive vaccination benefits as more positive, and accordingly, tended to present higher levels of adolescents’ vaccination adherence. According to Szilagy et al., a key trusted source of information regarding COVID-19 vaccines for children was the pediatrician [45]. Unlike other studies [14,15,17,19,54], we did not find a direct link between the parents’ COVID-19 perceived threat and their willingness to vaccinate their adolescents. However, a recent comprehensive literature review supports the current study’s finding, as it did not identify the perceived threat as one of the major factors affecting vaccine uptake [55].

The current study highlights the importance of the perceived benefits in promoting COVID-19 vaccine uptake. This was also discussed by Robertson et al., who found that hesitant participants differ from accepting participants more in how they perceived the benefits of vaccination than in how they perceived the vaccine risks [56]. Understanding the pivotal role of the vaccinations’ perceived benefits may have an applied implication, highlighting the need to address parents and raise their awareness of the benefits of vaccination, while at the same time, addressing their barriers and concerns. According to our results, the perceived benefits act as a mediator in the association between the perceived threat and vaccination, as well as in the association between trust in the healthcare system and vaccination. A higher perception of threat was found to be associated with perceiving the vaccination benefits as more prevalent and positive, which, in turn, was associated with enhanced adolescents’ vaccination adherence.

The current study also attempted to examine the importance of adolescents’ involvement in the vaccination decision-making process. Our findings show that parents’ perception of their adolescents’ level of involvement in this decision was average, and was significantly associated with actual vaccination. This should be of no surprise, as the current study population includes parents of adolescents aged 16–18 years, very close to an age when they are expected to make decisions for themselves regarding their health. Studies addressing this subject show that adolescents believe they should make vaccination decisions for themselves [14], and when information is provided, they tend to present high levels of willingness to become vaccinated [54]. This finding emphasizes the fact that while it is important to target parents when planning interventions, addressing adolescents directly should also be considered, to further promote vaccination.

Finally, as found in other studies examining COVID-19 vaccine compliance [57,58], the current study suggests that adolescents’ COVID-19 vaccination compliance may vary according to their parents’ socio-demographic background. The current study’s results suggest that the likelihood of adolescent vaccination is lower for parents with more children in the family [58,59]. In Israeli society, this may point at populations with many children as “at risk” populations, to whom more attention should be paid.

The current study may be subject to several limitations. The study was conducted in Israel and its results may not fully reflect other countries with less access to vaccines or different circumstances. Conducting studies that include various countries may help clarify deep-routed aspects associated with vaccine hesitancy and adherence. This study may also be subjected to selection bias as it is based on a self-reporting method. To minimize such bias, data collection was performed by a well-established survey company with a large respondent pool. This company takes comprehensive measures to reduce selection bias by using quota sampling and restricting the number on times one can take part in a survey during a defined period. Due to its cross-sectional design, this study does not enable causal inferences among the study variables. Moreover, the study population included the Israeli Jewish population only; therefore, our results may not reflect minority groups, such as Israeli Arabs (Palestinians). It would be interesting to examine these aspects among Israeli minority groups, as adherence rates to the vaccine among these groups were significantly lower as compared with the majority population. Moreover, adolescents’ involvement was assessed by their parents. As such, it may not fully reflect the adolescents’ actual views. Finally, this study was designed and carried out before the Delta and Omicron waves. New strains of the disease cause new morbidity waves with varying degrees of severity. However, more and more studies show that vaccines are effective in reducing disease severity and are safe for all age groups.

## 5. Conclusions

Promoting parents’ trust and addressing vaccination benefits and barriers are essential, primarily emphasizing the vaccines as enabling adolescents to resume their pre-COVID-19 routine, while reducing concerns regarding the vaccine’s safety and long-term effects. Adolescents should be regarded as partners in making vaccination decisions, providing them with information and enhancing their motivation to get vaccinated. These recommendations might be applied when interventions to improve vaccination adherence are designed. For example, parents and adolescents should be considered as separate target populations, and subjects, such as trust in the healthcare system, as well as benefits of the vaccine, should be incorporated and even highlighted. These issues should also be addressed when public health media campaigns are designed, pinpointing the messages according to the audience (parents or adolescents). These two strategies may, on one hand, help promote parents’ adherence, as well as accepting their adolescents as relevant in the vaccination decision, and on the other hand, help engage adolescents to take a more active role in the vaccination decision.

## Figures and Tables

**Table 1:** Means, standard deviations, and intercorrelations for the independent variables (*n* = 581).

	M (SD)	2.	3.	4.	5.	6.	7.	8.	9.	10.	11.
1. Parent age (34–71)	47.61 (5.01)	−0.26 *	−0.03	−0.03	0.03	0.01	0.02	0.09	0.11	-0.08	0.05
2. Number of children (1–13)	3.35 (1.60)		−0.02	0.18 *	0.02	−0.01	−0.08	−0.17 *	−0.06	0.01	−0.08
3. Education (0–1)	0.53 (0.50)			−0.19 *	−0.02	0.03	−0.14 *	−0.06	0.02	−0.10	−0.11
4. COVID-19 sickness (0–1)	0.26 (0.44)				0.05	−0.02	−0.04	−0.03	0.03	−0.01	−0.02
5. Childhood vaccinations (0–3)	1.83 (0.87)					0.26 *	0.13	0.23 *	0.34 *	−0.18 *	−0.05
6. Trust (1–7)	5.17 (1.19)						0.20 *	0.33 *	0.48 *	−0.29 *	0.04
7. Parent behavior (1–5)	3.76 (0.81)							0.38 *	0.24 *	−0.06	0.01
8. Percieved threat (1–5)	3.65 (0.95)								0.56 *	−0.02	0.05
9. Percieved benefits (1–5)	3.67 (0.89)									−0.37 *	0.06
10. Percieved barriers (1–5)	3.07 (1.01)										−0.07
11. Adolescent involvement (0–10)	6.26 (3.16)										

* *p* < 0.001, Bonferroni adjustment for multiple comparisons was applied. Note. Education level: academic—1 vs. nonacademic—0; COVID-19 sickness by parent or a family member: yes—1, no—0; childhood vaccinations: sum of routine vaccines, HPV vaccination, influenza vaccination (yes—1, no—0 for each); trust: in healthcare system; parents’ behavior: compliance regarding themselves and the adolescent; perceived COVID threat, benefits and barriers to COVID-19 vaccination: HBM theory; adolescent’s involvement in the decision to vaccinate: full—10, none—0.

**Table 2:** Logistic regression model for adolescent vaccination against COVID-19 with background variables, HBM variables, and adolescent’s involvement in the decision-making process (*n* = 581).

	*B*	*SeB*	OR (95%CI)	*p*
Step 1				
Parent age	0.01	0.03	1.01 (0.94, 1.07)	0.850
Number of children	−0.89	0.40	0.41 (0.19, 0.89)	0.025
Education	0.31	0.30	1.37 (0.77, 2.44)	0.289
COVID-19 sickness	0.13	0.33	1.14 (0.60, 2.18)	0.694
Step 2				
Childhood vaccinations	0.34	0.17	1.41 (1.01, 1.95)	0.042
Trust	0.01	0.14	1.01 (0.77, 1.33)	0.951
Parent behavior	0.07	0.15	1.08 (0.80, 1.45)	0.621
Step 3				
Percieved threat	0.21	0.20	1.23 (0.83, 1.81)	0.300
Percieved Benefits	1.86	0.26	6.42 (3.85, 10.69)	< 0.001
Percieved Barriers	−0.71	0.18	0.49 (0.35, 0.70)	< 0.001
Step 4				
Adolescent involvement	0.09	0.04	1.09 (1.01, 1.18)	0.021

Note. Step 1: χ^2^(4) = 15.13, *p* = 0.004, Nagelkerke’s R^2^ = 0.041; Step 2: χ^2^(3) = 93.16, *p* < 0.001, Nagelkerke’s ΔR^2^ = 0.227; Step 3: χ^2^(3) = 152.85, *p* < 0.001, Nagelkerke’s ΔR^2^ = 0.300; Step 4: χ^2^(1) = 5.34, *p* = 0.021, Nagelkerke’s ΔR^2^ = 0.011; Total model: χ^2^(11) = 264.12, *p* < 0.001, Nagelkerke’s R^2^ = 0.579.

## Data Availability

The datasets used and/or analyzed during the current study are available from the corresponding author upon reasonable request, and subject to YVC permission.
